# Supplementary figures and images for: IFNγ regulates MR1 transcription and antigen presentation
Source: Front Immunol. 2025 Sep 26;16:1624767. doi: 10.3389/fimmu.2025.1624767 (PMC12510863; doi:10.3389/fimmu.2025.1624767)

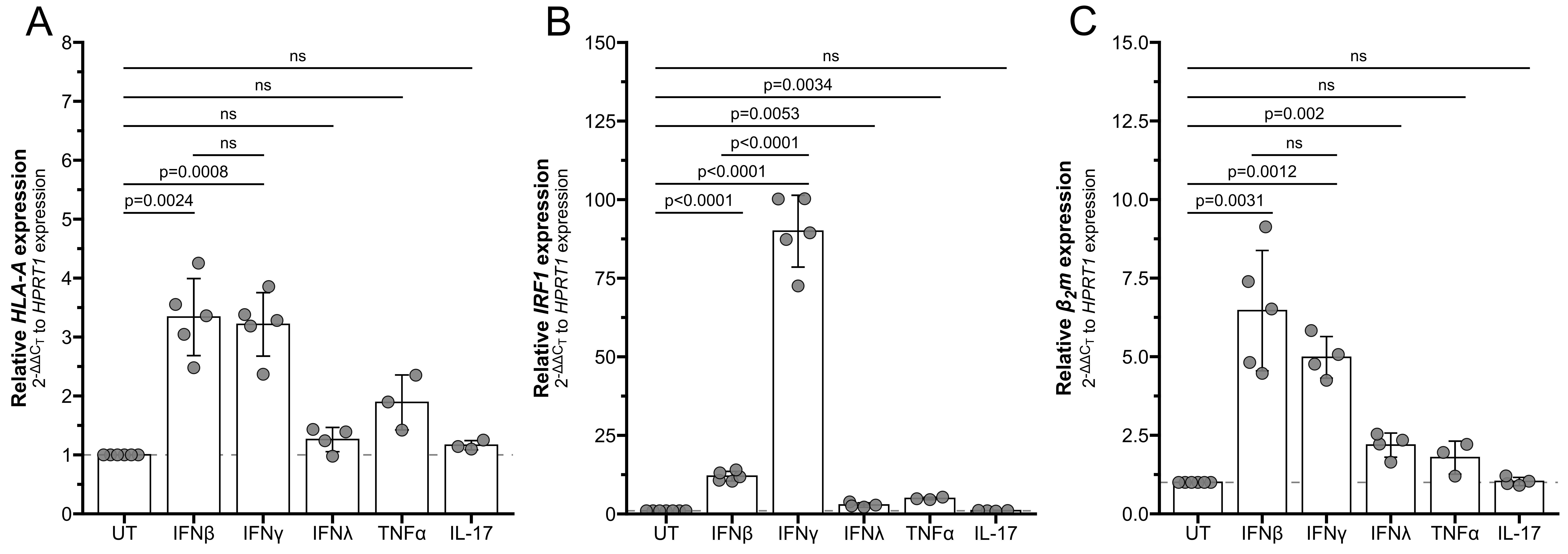

Supplement: Supplementary Figure 4 — Associated with Figure 7 . (A-C) RT-qPCR of wildtype BEAS-2B cells treated with recombinant human cytokines for 12 hours. Gene expression was calculated relative to HPRT1 expression and UT controls. Statistical analyses are in Supplementary Table 5 . [file Image4.tif]
