# Supplementary material for: IFNγ regulates MR1 transcription and antigen presentation
Source: Front Immunol. 2025 Sep 26;16:1624767. doi: 10.3389/fimmu.2025.1624767 (PMC12510863; doi:10.3389/fimmu.2025.1624767)
Supplement: Supplementary file 5 [file Table1.docx]

**Supplementary Table 1 Statistics associated with Figure 1 and Supplemental Figure 1.**

| Figure | Data | **Sample 1** | | | **Sample 2** | | | n1 | n2 | df | statistic | p-value | sig. |
| --- | --- | --- | --- | --- | --- | --- | --- | --- | --- | --- | --- | --- | --- |
|  |  | Cell | Ag | T cell | Cell | Ag | T cell |  |  |  |  |  |  |
| 1A, SF1A | *MR1* mRNA | AEC | UI | NT | AEC | *Sp* | NT | 5 | 5 | 4 | 1.913 | 0.1283 | ns |
| 1A, SF1A | *MR1* mRNA | AEC | UI | NT | AEC | UI | MAIT | 5 | 5 | 4 | 1.702 | 0.1639 | ns |
| 1A, SF1A | *MR1* mRNA | AEC | UI | NT | AEC | *Sp* | MAIT | 5 | 5 | 4 | 8.332 | 0.0011 | ** |
| 1A, SF1A | *MR1* mRNA | AEC | *Sp* | NT | AEC | UI | MAIT | 5 | 5 | 4 | 0.391 | 0.7157 | ns |
| 1A, SF1A | *MR1* mRNA | AEC | *Sp* | NT | AEC | *Sp* | MAIT | 5 | 5 | 4 | 3.679 | 0.0212 | * |
| 1A, SF1A | *MR1* mRNA | AEC | UI | MAIT | AEC | *Sp* | MAIT | 5 | 5 | 4 | 2.846 | 0.0466 | * |
| 1B, SF1B | *MR1* mRNA | B2B | UI | NT | B2B | *Ms* | NT | 3 | 3 | 2 | 1.387 | 0.2998 | ns |
| 1B, SF1B | *MR1* mRNA | B2B | UI | NT | B2B | UI | MAIT | 3 | 3 | 2 | 1.653 | 0.24 | ns |
| 1B, SF1B | *MR1* mRNA | B2B | UI | NT | B2B | *Ms* | MAIT | 3 | 3 | 2 | 3.689 | 0.0663 | ns |
| 1B, SF1B | *MR1* mRNA | B2B | *Ms* | NT | B2B | UI | MAIT | 3 | 3 | 2 | 2.253 | 0.153 | ns |
| 1B, SF1B | *MR1* mRNA | B2B | *Ms* | NT | B2B | *Ms* | MAIT | 3 | 3 | 2 | 3.702 | 0.0658 | ns |
| 1B, SF1B | *MR1* mRNA | B2B | UI | MAIT | B2B | *Ms* | MAIT | 3 | 3 | 2 | 3.095 | 0.0905 | ns |
| 1C, SF1C | αMR1 gMFI | B2B | UI | NT | B2B | *Ms* | NT | 3 | 3 | 2 | 1.31 | 0.3205 | ns |
| 1C, SF1C | αMR1 gMFI | B2B | UI | NT | B2B | UI | MAIT | 3 | 3 | 2 | 0.8576 | 0.4815 | ns |
| 1C, SF1C | αMR1 gMFI | B2B | UI | NT | B2B | *Ms* | MAIT | 3 | 3 | 2 | 15.02 | 0.0044 | ** |
| 1C, SF1C | αMR1 gMFI | B2B | *Ms* | NT | B2B | UI | MAIT | 3 | 3 | 2 | 0.7913 | 0.5117 | ns |
| 1C, SF1C | αMR1 gMFI | B2B | *Ms* | NT | B2B | *Ms* | MAIT | 3 | 3 | 2 | 12.17 | 0.0067 | ** |
| 1C, SF1C | αMR1 gMFI | B2B | UI | MAIT | B2B | *Ms* | MAIT | 3 | 3 | 2 | 5.032 | 0.0373 | * |
| 1D, SF1D | MR1 mRNA | B2B | UT | NT | B2B | 5-OP | NT | 3 | 3 | 2 | 0.8515 | 0.4842 | ns |
| 1D, SF1D | MR1 mRNA | B2B | UT | NT | B2B | UT | MAIT | 3 | 3 | 2 | 1.653 | 0.24 | ns |
| 1D, SF1D | MR1 mRNA | B2B | UT | NT | B2B | 5-OP | MAIT | 3 | 3 | 2 | 4.931 | 0.0388 | * |
| 1D, SF1D | MR1 mRNA | B2B | 5-OP | NT | B2B | UT | MAIT | 3 | 3 | 2 | 1.635 | 0.2437 | ns |
| 1D, SF1D | MR1 mRNA | B2B | 5-OP | NT | B2B | 5-OP | MAIT | 3 | 3 | 2 | 4.784 | 0.041 | * |
| 1D, SF1D | MR1 mRNA | B2B | UT | MAIT | B2B | 5-OP | MAIT | 3 | 3 | 2 | 5.377 | 0.0329 | * |
| 1D, SF1D | MR1 mRNA | B2B | UT | NT | B2B | 6-FP | NT | 3 | 3 | 2 | 0.7058 | 0.5535 | ns |
| 1D, SF1D | MR1 mRNA | B2B | UT | NT | B2B | 6-FP | MAIT | 3 | 3 | 2 | 1.585 | 0.2539 | ns |
| 1D, SF1D | MR1 mRNA | B2B | 6-FP | NT | B2B | UT | MAIT | 3 | 3 | 2 | 1.318 | 0.3182 | ns |
| 1D, SF1D | MR1 mRNA | B2B | 6-FP | NT | B2B | 6-FP | MAIT | 3 | 3 | 2 | 1.466 | 0.2802 | ns |
| 1D, SF1D | MR1 mRNA | B2B | UT | MAIT | B2B | 6-FP | MAIT | 3 | 3 | 2 | 0.8054 | 0.5051 | ns |
| 1D, SF1D | MR1 mRNA | B2B | 5-OP | NT | B2B | 6-FP | NT | 3 | 3 | 2 | 0.5185 | 0.6557 | ns |
| 1D, SF1D | MR1 mRNA | B2B | 5-OP | MAIT | B2B | 6-FP | MAIT | 3 | 3 | 2 | 5.239 | 0.0346 | * |
| 1E, SF1E | αMR1 gMFI | B2B | UT | NT | B2B | 5-OP | NT | 4 | 4 | 3 | 1.312 | 0.2808 | ns |
| 1E, SF1E | αMR1 gMFI | B2B | UT | NT | B2B | UT | MAIT | 4 | 4 | 3 | 0.9286 | 0.4216 | ns |
| 1E, SF1E | αMR1 gMFI | B2B | UT | NT | B2B | 5-OP | MAIT | 4 | 4 | 3 | 2.826 | 0.0664 | ns |
| 1E, SF1E | αMR1 gMFI | B2B | 5-OP | NT | B2B | UT | MAIT | 4 | 4 | 3 | 2.042 | 0.1338 | ns |
| 1E, SF1E | αMR1 gMFI | B2B | 5-OP | NT | B2B | 5-OP | MAIT | 4 | 4 | 3 | 3.166 | 0.0506 | ns |
| 1E, SF1E | αMR1 gMFI | B2B | UT | MAIT | B2B | 5-OP | MAIT | 4 | 4 | 3 | 3.218 | 0.0487 | * |
| 1E, SF1E | αMR1 gMFI | B2B | UT | NT | B2B | 6-FP | NT | 4 | 4 | 3 | 0.2705 | 0.8043 | ns |
| 1E, SF1E | αMR1 gMFI | B2B | UT | NT | B2B | 6-FP | MAIT | 4 | 4 | 3 | 0.5864 | 0.5988 | ns |
| 1E, SF1E | αMR1 gMFI | B2B | 6-FP | NT | B2B | UT | MAIT | 4 | 4 | 3 | 1.558 | 0.2171 | ns |
| 1E, SF1E | αMR1 gMFI | B2B | 6-FP | NT | B2B | 6-FP | MAIT | 4 | 4 | 3 | 1.349 | 0.2703 | ns |
| 1E, SF1E | αMR1 gMFI | B2B | UT | MAIT | B2B | 6-FP | MAIT | 4 | 4 | 3 | 0.03772 | 0.9723 | ns |
| 1E, SF1E | αMR1 gMFI | B2B | 5-OP | NT | B2B | 6-FP | NT | 4 | 4 | 3 | 2.235 | 0.1114 | ns |
| 1E, SF1E | αMR1 gMFI | B2B | 5-OP | MAIT | B2B | 6-FP | MAIT | 4 | 4 | 3 | 2.559 | 0.0833 | ns |

*Definition of abbreviations:*

Ag = antigen; df = degrees of freedom; statistic = absolute value of T statistic; AEC = airway epithelial cells; B2B = BEAS-2B cells; UI = uninfected control; *Sp* = *Streptococcus pneumoniae*; *Ms* = *Mycobacterium smegmatis*; UT = media treated control; 5-OP = 5-OP-RU; 6-FP = 6-formylpterin; NT = no T cell control; gMFI = geometric mean fluorescence intensity. Sig: **** for p<0.0001; *** for 0.0001<p< 0.001; ** for 0.001<p< 0.01; * for 0.01<p< 0.05; ns for p>0.05.
