# Supplementary material for: IFNγ regulates MR1 transcription and antigen presentation
Source: Front Immunol. 2025 Sep 26;16:1624767. doi: 10.3389/fimmu.2025.1624767 (PMC12510863; doi:10.3389/fimmu.2025.1624767)
Supplement: Supplementary file 6 [file Table2.docx]

**Supplementary Table 2 Statistics associated with Figure 4 and Supplemental Figure 2.**

| Fig. | Data | **Sample 1** | | | **Sample 2** | | | n1 | n2 | df | statistic | p-value | sig. |
| --- | --- | --- | --- | --- | --- | --- | --- | --- | --- | --- | --- | --- | --- |
|  |  | NLRC5 | IRF1 | IFNγ | NLRC5 | IRF1 | IFNγ |  |  |  |  |  |  |
| 4A | *MR1* mRNA | Mis | Mis | UT | Mis | KD | UT | 3 | 3 | 2 | 0.6683 | 0.5727 | ns |
| 4A | *MR1* mRNA | Mis | Mis | UT | KD | Mis | UT | 3 | 3 | 2 | 0.5922 | 0.6138 | ns |
| 4A | *MR1* mRNA | Mis | Mis | UT | KD | KD | UT | 3 | 3 | 2 | 1.195 | 0.3545 | ns |
| 4A | *MR1* mRNA | Mis | Mis | UT | Mis | Mis | IFNγ | 3 | 3 | 2 | 36.62 | 0.0007 | *** |
| 4A | *MR1* mRNA | Mis | KD | UT | KD | Mis | UT | 3 | 3 | 2 | 1.517 | 0.2684 | ns |
| 4A | *MR1* mRNA | Mis | KD | UT | KD | KD | UT | 3 | 3 | 2 | 2.281 | 0.1501 | ns |
| 4A | *MR1* mRNA | Mis | KD | UT | Mis | KD | IFNγ | 3 | 3 | 2 | 5.223 | 0.0348 | * |
| 4A | *MR1* mRNA | KD | Mis | UT | KD | KD | UT | 3 | 3 | 2 | 3.215 | 0.0846 | ns |
| 4A | *MR1* mRNA | KD | Mis | UT | KD | Mis | IFNγ | 3 | 3 | 2 | 12.33 | 0.0065 | ** |
| 4A | *MR1* mRNA | KD | KD | UT | KD | KD | IFNγ | 3 | 3 | 2 | 14.32 | 0.0048 | ** |
| 4A | *MR1* mRNA | Mis | Mis | IFNγ | Mis | KD | IFNγ | 3 | 3 | 2 | 11.71 | 0.0072 | ** |
| 4A | *MR1* mRNA | Mis | Mis | IFNγ | KD | Mis | IFNγ | 3 | 3 | 2 | 1.156 | 0.3671 | ns |
| 4A | *MR1* mRNA | Mis | Mis | IFNγ | KD | KD | IFNγ | 3 | 3 | 2 | 17.41 | 0.0033 | ** |
| 4A | *MR1* mRNA | Mis | KD | IFNγ | KD | Mis | IFNγ | 3 | 3 | 2 | 6.393 | 0.0236 | * |
| 4A | *MR1* mRNA | Mis | KD | IFNγ | KD | KD | IFNγ | 3 | 3 | 2 | 3.15 | 0.0877 | ns |
| 4A | *MR1* mRNA | KD | Mis | IFNγ | KD | KD | IFNγ | 3 | 3 | 2 | 9.035 | 0.012 | * |
| SF2A | *IRF1* mRNA | Mis | Mis | UT | Mis | KD | UT | 3 | 3 | 2 | 8.304 | 0.0142 | * |
| SF2A | *IRF1* mRNA | Mis | Mis | UT | KD | Mis | UT | 3 | 3 | 2 | 0.7416 | 0.5356 | ns |
| SF2A | *IRF1* mRNA | Mis | Mis | UT | KD | KD | UT | 3 | 3 | 2 | 16.04 | 0.0039 | ** |
| SF2A | *IRF1* mRNA | Mis | Mis | UT | Mis | Mis | IFNγ | 3 | 3 | 2 | 10.94 | 0.0083 | ** |
| SF2A | *IRF1* mRNA | Mis | KD | UT | KD | Mis | UT | 3 | 3 | 2 | 5.412 | 0.0325 | * |
| SF2A | *IRF1* mRNA | Mis | KD | UT | KD | KD | UT | 3 | 3 | 2 | 0.8864 | 0.4689 | ns |
| SF2A | *IRF1* mRNA | Mis | KD | UT | Mis | KD | IFNγ | 3 | 3 | 2 | 3.729 | 0.065 | ns |
| SF2A | *IRF1* mRNA | KD | Mis | UT | KD | KD | UT | 3 | 3 | 2 | 8.973 | 0.0122 | * |
| SF2A | *IRF1* mRNA | KD | Mis | UT | KD | Mis | IFNγ | 3 | 3 | 2 | 4.914 | 0.039 | * |
| SF2A | *IRF1* mRNA | KD | KD | UT | KD | KD | IFNγ | 3 | 3 | 2 | 3.78 | 0.0634 | ns |
| SF2A | *IRF1* mRNA | Mis | Mis | IFNγ | Mis | KD | IFNγ | 3 | 3 | 2 | 15.06 | 0.0044 | ** |
| SF2A | *IRF1* mRNA | Mis | Mis | IFNγ | KD | Mis | IFNγ | 3 | 3 | 2 | 0.8739 | 0.4743 | ns |
| SF2A | *IRF1* mRNA | Mis | Mis | IFNγ | KD | KD | IFNγ | 3 | 3 | 2 | 17.23 | 0.0034 | ** |
| SF2A | *IRF1* mRNA | Mis | KD | IFNγ | KD | Mis | IFNγ | 3 | 3 | 2 | 4.394 | 0.0481 | * |
| SF2A | *IRF1* mRNA | Mis | KD | IFNγ | KD | KD | IFNγ | 3 | 3 | 2 | 0.9636 | 0.4369 | ns |
| SF2A | *IRF1* mRNA | KD | Mis | IFNγ | KD | KD | IFNγ | 3 | 3 | 2 | 5.308 | 0.0337 | * |
| SF2B | *NLRC5* mRNA | Mis | Mis | UT | Mis | KD | UT | 3 | 3 | 2 | 14.19 | 0.0049 | ** |
| SF2B | *NLRC5* mRNA | Mis | Mis | UT | KD | Mis | UT | 3 | 3 | 2 | 1.149 | 0.3696 | ns |
| SF2B | *NLRC5* mRNA | Mis | Mis | UT | KD | KD | UT | 3 | 3 | 2 | 2.041 | 0.178 | ns |
| SF2B | *NLRC5* mRNA | Mis | Mis | UT | Mis | Mis | IFNγ | 3 | 3 | 2 | 8.078 | 0.015 | * |
| SF2B | *NLRC5* mRNA | Mis | KD | UT | KD | Mis | UT | 3 | 3 | 2 | 0.04497 | 0.9682 | ns |
| SF2B | *NLRC5* mRNA | Mis | KD | UT | KD | KD | UT | 3 | 3 | 2 | 0.7383 | 0.5372 | ns |
| SF2B | *NLRC5* mRNA | Mis | KD | UT | Mis | KD | IFNγ | 3 | 3 | 2 | 5.053 | 0.037 | * |
| SF2B | *NLRC5* mRNA | KD | Mis | UT | KD | KD | UT | 3 | 3 | 2 | 1.568 | 0.2575 | ns |
| SF2B | *NLRC5* mRNA | KD | Mis | UT | KD | Mis | IFNγ | 3 | 3 | 2 | 4.443 | 0.0471 | * |
| SF2B | *NLRC5* mRNA | KD | KD | UT | KD | KD | IFNγ | 3 | 3 | 2 | 15.64 | 0.0041 | ** |
| SF2B | *NLRC5* mRNA | Mis | Mis | IFNγ | Mis | KD | IFNγ | 3 | 3 | 2 | 1.171 | 0.3621 | ns |
| SF2B | *NLRC5* mRNA | Mis | Mis | IFNγ | KD | Mis | IFNγ | 3 | 3 | 2 | 2.113 | 0.1689 | ns |
| SF2B | *NLRC5* mRNA | Mis | Mis | IFNγ | KD | KD | IFNγ | 3 | 3 | 2 | 2.631 | 0.1192 | ns |
| SF2B | *NLRC5* mRNA | Mis | KD | IFNγ | KD | Mis | IFNγ | 3 | 3 | 2 | 5.198 | 0.0351 | * |
| SF2B | *NLRC5* mRNA | Mis | KD | IFNγ | KD | KD | IFNγ | 3 | 3 | 2 | 1.353 | 0.3087 | ns |
| SF2B | *NLRC5* mRNA | KD | Mis | IFNγ | KD | KD | IFNγ | 3 | 3 | 2 | 0.1013 | 0.9286 | ns |
| 4B | *MR1* mRNA | Cas9 | Mis | UT | Cas9 | KD | UT | 3 | 3 | 2 | 1.281 | 0.3286 | ns |
| 4B | *MR1* mRNA | Cas9 | Mis | UT | KO #1 | Mis | UT | 3 | 3 | 2 | N/A | N/A | N/A |
| 4B | *MR1* mRNA | Cas9 | Mis | UT | KO #1 | KD | UT | 3 | 3 | 2 | 4.524 | 0.0456 | * |
| 4B | *MR1* mRNA | Cas9 | Mis | UT | Cas9 | Mis | IFNγ | 3 | 3 | 2 | 8.226 | 0.0145 | * |
| 4B | *MR1* mRNA | Cas9 | KD | UT | KO #1 | Mis | UT | 3 | 3 | 2 | 1.281 | 0.3286 | ns |
| 4B | *MR1* mRNA | Cas9 | KD | UT | KO #1 | KD | UT | 3 | 3 | 2 | 1.352 | 0.3089 | ns |
| 4B | *MR1* mRNA | Cas9 | KD | UT | Cas9 | KD | IFNγ | 3 | 3 | 2 | 4.923 | 0.0389 | * |
| 4B | *MR1* mRNA | KO #1 | Mis | UT | KO #1 | KD | UT | 3 | 3 | 2 | 4.524 | 0.0456 | * |
| 4B | *MR1* mRNA | KO #1 | Mis | UT | KO #1 | Mis | IFNγ | 3 | 3 | 2 | 42.26 | 0.0006 | *** |
| 4B | *MR1* mRNA | KO #1 | KD | UT | KO #1 | KD | IFNγ | 3 | 3 | 2 | 1.525 | 0.2667 | ns |
| 4B | *MR1* mRNA | Cas9 | Mis | IFNγ | Cas9 | KD | IFNγ | 3 | 3 | 2 | 4.489 | 0.0462 | * |
| 4B | *MR1* mRNA | Cas9 | Mis | IFNγ | KO #1 | Mis | IFNγ | 3 | 3 | 2 | 1.778 | 0.2173 | ns |
| 4B | *MR1* mRNA | Cas9 | Mis | IFNγ | KO #1 | KD | IFNγ | 3 | 3 | 2 | 2.469 | 0.1323 | ns |
| 4B | *MR1* mRNA | Cas9 | KD | IFNγ | KO #1 | Mis | IFNγ | 3 | 3 | 2 | 6.989 | 0.0199 | * |
| 4B | *MR1* mRNA | Cas9 | KD | IFNγ | KO #1 | KD | IFNγ | 3 | 3 | 2 | 0.01689 | 0.9881 | ns |
| 4B | *MR1* mRNA | KO #1 | Mis | IFNγ | KO #1 | KD | IFNγ | 3 | 3 | 2 | 2.643 | 0.1183 | ns |
| 4C | *HLA-A* mRNA | Cas9 | Mis | UT | Cas9 | KD | UT | 3 | 3 | 2 | 0.5836 | 0.6186 | ns |
| 4C | *HLA-A* mRNA | Cas9 | Mis | UT | KO #1 | Mis | UT | 3 | 3 | 2 | N/A | N/A | N/A |
| 4C | *HLA-A* mRNA | Cas9 | Mis | UT | KO #1 | KD | UT | 3 | 3 | 2 | 4.16 | 0.0532 | ns |
| 4C | *HLA-A* mRNA | Cas9 | Mis | UT | Cas9 | Mis | IFNγ | 3 | 3 | 2 | 4.282 | 0.0504 | ns |
| 4C | *HLA-A* mRNA | Cas9 | KD | UT | KO #1 | Mis | UT | 3 | 3 | 2 | 0.5836 | 0.6186 | ns |
| 4C | *HLA-A* mRNA | Cas9 | KD | UT | KO #1 | KD | UT | 3 | 3 | 2 | 2.37 | 0.1412 | ns |
| 4C | *HLA-A* mRNA | Cas9 | KD | UT | Cas9 | KD | IFNγ | 3 | 3 | 2 | 6.746 | 0.0213 | * |
| 4C | *HLA-A* mRNA | KO #1 | Mis | UT | KO #1 | KD | UT | 3 | 3 | 2 | 4.16 | 0.0532 | ns |
| 4C | *HLA-A* mRNA | KO #1 | Mis | UT | KO #1 | Mis | IFNγ | 3 | 3 | 2 | 6.47 | 0.0231 | * |
| 4C | *HLA-A* mRNA | KO #1 | KD | UT | KO #1 | KD | IFNγ | 3 | 3 | 2 | 3.794 | 0.063 | ns |
| 4C | *HLA-A* mRNA | Cas9 | Mis | IFNγ | Cas9 | KD | IFNγ | 3 | 3 | 2 | 3.145 | 0.088 | ns |
| 4C | *HLA-A* mRNA | Cas9 | Mis | IFNγ | KO #1 | Mis | IFNγ | 3 | 3 | 2 | 3.393 | 0.077 | ns |
| 4C | *HLA-A* mRNA | Cas9 | Mis | IFNγ | KO #1 | KD | IFNγ | 3 | 3 | 2 | 3.575 | 0.0701 | ns |
| 4C | *HLA-A* mRNA | Cas9 | KD | IFNγ | KO #1 | Mis | IFNγ | 3 | 3 | 2 | 0.2266 | 0.8418 | ns |
| 4C | *HLA-A* mRNA | Cas9 | KD | IFNγ | KO #1 | KD | IFNγ | 3 | 3 | 2 | 4.039 | 0.0562 | ns |
| 4C | *HLA-A* mRNA | KO #1 | Mis | IFNγ | KO #1 | KD | IFNγ | 3 | 3 | 2 | 3.875 | 0.0606 | ns |
| SF2C | *IRF1* mRNA | Cas9 | Mis | UT | Cas9 | KD | UT | 3 | 3 | 2 | 5.042 | 0.0372 | * |
| SF2C | *IRF1* mRNA | Cas9 | Mis | UT | KO #1 | Mis | UT | 3 | 3 | 2 | N/A | N/A | N/A |
| SF2C | *IRF1* mRNA | Cas9 | Mis | UT | KO #1 | KD | UT | 3 | 3 | 2 | 6.233 | 0.0248 | * |
| SF2C | *IRF1* mRNA | Cas9 | Mis | UT | Cas9 | Mis | IFNγ | 3 | 3 | 2 | 16.02 | 0.0039 | ** |
| SF2C | *IRF1* mRNA | Cas9 | KD | UT | KO #1 | Mis | UT | 3 | 3 | 2 | 5.042 | 0.0372 | * |
| SF2C | *IRF1* mRNA | Cas9 | KD | UT | KO #1 | KD | UT | 3 | 3 | 2 | 0.1761 | 0.8764 | ns |
| SF2C | *IRF1* mRNA | Cas9 | KD | UT | Cas9 | KD | IFNγ | 3 | 3 | 2 | 2.58 | 0.1231 | ns |
| SF2C | *IRF1* mRNA | KO #1 | Mis | UT | KO #1 | KD | UT | 3 | 3 | 2 | 6.233 | 0.0248 | * |
| SF2C | *IRF1* mRNA | KO #1 | Mis | UT | KO #1 | Mis | IFNγ | 3 | 3 | 2 | 15.4 | 0.0042 | ** |
| SF2C | *IRF1* mRNA | KO #1 | KD | UT | KO #1 | KD | IFNγ | 3 | 3 | 2 | 2.362 | 0.1420 | ns |
| SF2C | *IRF1* mRNA | Cas9 | Mis | IFNγ | Cas9 | KD | IFNγ | 3 | 3 | 2 | 6.101 | 0.0258 | * |
| SF2C | *IRF1* mRNA | Cas9 | Mis | IFNγ | KO #1 | Mis | IFNγ | 3 | 3 | 2 | 2.222 | 0.1564 | ns |
| SF2C | *IRF1* mRNA | Cas9 | Mis | IFNγ | KO #1 | KD | IFNγ | 3 | 3 | 2 | 5.922 | 0.0273 | * |
| SF2C | *IRF1* mRNA | Cas9 | KD | IFNγ | KO #1 | Mis | IFNγ | 3 | 3 | 2 | 7.389 | 0.0178 | * |
| SF2C | *IRF1* mRNA | Cas9 | KD | IFNγ | KO #1 | KD | IFNγ | 3 | 3 | 2 | 1.52 | 0.2679 | ns |
| SF2C | *IRF1* mRNA | KO #1 | Mis | IFNγ | KO #1 | KD | IFNγ | 3 | 3 | 2 | 7.82 | 0.0160 | * |
| 4D | IFNγ SFU | Cas9 | Mis | IFNγ | Cas9 | KD | IFNγ | 3 | 3 | 2.653 | 5.207 | 0.0186 | * |
| 4D | IFNγ SFU | Cas9 | Mis | IFNγ | KO #1 | Mis | IFNγ | 3 | 3 | 3.03 | 0.08493 | 0.9376 | ns |
| 4D | IFNγ SFU | Cas9 | Mis | IFNγ | KO #1 | KD | IFNγ | 3 | 3 | 3.95 | 9.49725 | 0.0007 | *** |
| 4D | IFNγ SFU | Cas9 | KD | IFNγ | KO #1 | Mis | IFNγ | 3 | 3 | 3.772 | 4.5 | 0.0124 | * |
| 4D | IFNγ SFU | Cas9 | KD | IFNγ | KO #1 | KD | IFNγ | 3 | 3 | 2.525 | 0.3875 | 0.7287 | ns |
| 4D | IFNγ SFU | KO #1 | Mis | IFNγ | KO #1 | KD | IFNγ | 3 | 3 | 2.843 | 6.153 | 0.0101 | * |
| 4E | *MR1* mRNA | N/A | Cas9 | UT | N/A | Cas9 | IFNγ | 6 | 6 | 5 | 7.089 | 0.0009 | *** |
| 4E | *MR1* mRNA | N/A | KO #2 | UT | N/A | KO #2 | IFNγ | 6 | 6 | 5 | 1.049 | 0.3424 | ns |
| 4E | *MR1* mRNA | N/A | Cas9 | UT | N/A | KO #2 | UT | 6 | 6 | 5 | N/A | N/A | N/A |
| 4E | *MR1* mRNA | N/A | Cas9 | IFNγ | N/A | KO #2 | IFNγ | 6 | 6 | 5.718 | 5.664 | 0.0024 | ** |
| 4F | *HLA-A* mRNA | N/A | Cas9 | UT | N/A | Cas9 | IFNγ | 6 | 6 | 5 | 7.715 | 0.0006 | *** |
| 4F | *HLA-A* mRNA | N/A | KO #2 | UT | N/A | KO #2 | IFNγ | 6 | 6 | 5 | 1.094 | 0.3237 | ns |
| 4F | *HLA-A* mRNA | N/A | Cas9 | UT | N/A | KO #2 | UT | 6 | 6 | 5 | N/A | N/A | N/A |
| 4F | *HLA-A* mRNA | N/A | Cas9 | IFNγ | N/A | KO #2 | IFNγ | 6 | 6 | 7.874 | 10.31 | 0.0001 | *** |
| SF2F | *MR1* mRNA | N/A | Cas9 | UT | N/A | Cas9 | IFNγ | 4 | 4 | 3 | 12.35 | 0.0011 | ** |
| SF2F | *MR1* mRNA | N/A | KO #1 | UT | N/A | KO #1 | IFNγ | 4 | 4 | 3 | 12.78 | 0.001 | ** |
| SF2F | *MR1* mRNA | N/A | Cas9 | UT | N/A | KO #1 | UT | 4 | 4 | 3 | N/A | N/A | N/A |
| SF2F | *MR1* mRNA | N/A | Cas9 | IFNγ | N/A | KO #1 | IFNγ | 4 | 4 | 3.226 | 8.578 | 0.0033 | ** |
| 4G | αMR1 gMFI | N/A | Cas9 | UT | N/A | Cas9 | IFNγ | 4 | 4 | 3 | 5.082 | 0.0147 | * |
| 4G | αMR1 gMFI | N/A | KO #1 | UT | N/A | KO #1 | IFNγ | 4 | 4 | 3 | 0.5987 | 0.5916 | ns |
| 4G | αMR1 gMFI | N/A | Cas9 | UT | N/A | KO #1 | UT | 4 | 4 | 5.949 | 0.4949 | 0.6384 | ns |
| 4G | αMR1 gMFI | N/A | Cas9 | IFNγ | N/A | KO #1 | IFNγ | 4 | 4 | 5.97 | 0.7191 | 0.4992 | ns |
| 4H | αMHC-Ia gMFI | N/A | Cas9 | UT | N/A | Cas9 | IFNγ | 4 | 4 | 3 | 4.887 | 0.0164 | * |
| 4H | αMHC-Ia gMFI | N/A | KO #1 | UT | N/A | KO #1 | IFNγ | 4 | 4 | 3 | 1.184 | 0.3216 | ns |
| 4H | αMHC-Ia gMFI | N/A | Cas9 | UT | N/A | KO #1 | UT | 4 | 4 | 5.827 | 0.7061 | 0.5073 | ns |
| 4H | αMHC-Ia gMFI | N/A | Cas9 | IFNγ | N/A | KO #1 | IFNγ | 4 | 4 | 3.507 | 3.38 | 0.034 | * |
| SF2G | αMR1 gMFI | N/A | Cas9 | UT | N/A | Cas9 | IFNγ | 3 | 3 | 2 | 5.068 | 0.0368 | * |
| SF2G | αMR1 gMFI | N/A | KO #2 | UT | N/A | KO #2 | IFNγ | 3 | 3 | 2 | 1.736 | 0.2246 | ns |
| SF2G | αMR1 gMFI | N/A | Cas9 | UT | N/A | KO #2 | UT | 3 | 3 | 3.48 | 0.7566 | 0.4973 | ns |
| SF2G | αMR1 gMFI | N/A | Cas9 | IFNγ | N/A | KO #2 | IFNγ | 3 | 3 | 3.933 | 1.387 | 0.2389 | ns |
| SF2H | αMHC-Ia gMFI | N/A | Cas9 | UT | N/A | Cas9 | IFNγ | 3 | 3 | 2 | 4.391 | 0.0481 | * |
| SF2H | αMHC-Ia gMFI | N/A | KO #2 | UT | N/A | KO #2 | IFNγ | 3 | 3 | 2 | 0.04702 | 0.9668 | ns |
| SF2H | αMHC-Ia gMFI | N/A | Cas9 | UT | N/A | KO #2 | UT | 3 | 3 | 3.029 | 0.4856 | 0.6602 | ns |
| SF2H | αMHC-Ia gMFI | N/A | Cas9 | IFNγ | N/A | KO #2 | IFNγ | 3 | 3 | 2.597 | 2.543 | 0.0975 | ns |

*Definition of abbreviations:*

df = degrees of freedom; statistic = absolute value of T statistic; Mis = missense siRNA control; KD = siRNA gene knockdown; UT = media control; Cas9 = Cas9^+^ BEAS-2B control cells; KO = CRISPR gene knockout cell line; N/A = not applicable; gMFI = geometric mean fluorescence intensity. Sig: **** for p<0.0001; *** for 0.0001<p< 0.001; ** for 0.001<p< 0.01; * for 0.01<p< 0.05; ns for p>0.05.
