# Supplementary material for: IFNγ regulates MR1 transcription and antigen presentation
Source: Front Immunol. 2025 Sep 26;16:1624767. doi: 10.3389/fimmu.2025.1624767 (PMC12510863; doi:10.3389/fimmu.2025.1624767)
Supplement: Supplementary file 7 [file Table3.docx]

**Supplementary Table 3 Statistics associated with Figure 5, Figure 6, and Supplemental Figure 3.**

| Figure | Data | **Sample 1** | | | **Sample 2** | | | n1 | n2 | df | statistic | p-value | sig. |
| --- | --- | --- | --- | --- | --- | --- | --- | --- | --- | --- | --- | --- | --- |
|  |  | Cell | Ag | T cell | Cell | Ag | T cell |  |  |  |  |  |  |
| 5A, SF3A | pSTAT1 gMFI | AEC | UI | NT | AEC | *Sp* | NT | 3 | 3 | 2 | 1.018 | 0.4159 | ns |
| 5A, SF3A | pSTAT1 gMFI | AEC | UI | NT | AEC | UI | MAIT | 3 | 3 | 2 | 1.472 | 0.2788 | ns |
| 5A, SF3A | pSTAT1 gMFI | AEC | UI | NT | AEC | *Sp* | MAIT | 3 | 3 | 2 | 9.043 | 0.012 | * |
| 5A, SF3A | pSTAT1 gMFI | AEC | *Sp* | NT | AEC | UI | MAIT | 3 | 3 | 2 | 0.5673 | 0.6277 | ns |
| 5A, SF3A | pSTAT1 gMFI | AEC | *Sp* | NT | AEC | *Sp* | MAIT | 3 | 3 | 2 | 4.031 | 0.0564 | ns |
| 5A, SF3A | pSTAT1 gMFI | AEC | UI | MAIT | AEC | *Sp* | MAIT | 3 | 3 | 2 | 6.541 | 0.0226 | * |
| 5C, SF3B | *B2m* mRNA | AEC | UI | NT | AEC | *Sp* | NT | 4 | 4 | 3 | 3.014 | 0.057 | ns |
| 5C, SF3B | *B2m* mRNA | AEC | UI | NT | AEC | UI | MAIT | 4 | 4 | 3 | 4.013 | 0.0278 | * |
| 5C, SF3B | *B2m* mRNA | AEC | UI | NT | AEC | *Sp* | MAIT | 4 | 4 | 3 | 4.361 | 0.0223 | * |
| 5C, SF3B | *B2m* mRNA | AEC | *Sp* | NT | AEC | UI | MAIT | 4 | 4 | 3 | 0.9405 | 0.4164 | ns |
| 5C, SF3B | *B2m* mRNA | AEC | *Sp* | NT | AEC | *Sp* | MAIT | 4 | 4 | 3 | 4.302 | 0.0231 | * |
| 5C, SF3B | *B2m* mRNA | AEC | UI | MAIT | AEC | *Sp* | MAIT | 4 | 4 | 3 | 3.741 | 0.0333 | * |
| 5D, SF3D | *HLA-A* mRNA | AEC | UI | NT | AEC | *Sp* | NT | 5 | 5 | 4 | 2.918 | 0.0433 | * |
| 5D, SF3D | *HLA-A* mRNA | AEC | UI | NT | AEC | UI | MAIT | 5 | 5 | 4 | 2.629 | 0.0582 | ns |
| 5D, SF3D | *HLA-A* mRNA | AEC | UI | NT | AEC | *Sp* | MAIT | 5 | 5 | 4 | 2.925 | 0.043 | * |
| 5D, SF3D | *HLA-A* mRNA | AEC | *Sp* | NT | AEC | UI | MAIT | 5 | 5 | 4 | 1.346 | 0.2496 | ns |
| 5D, SF3D | *HLA-A* mRNA | AEC | *Sp* | NT | AEC | *Sp* | MAIT | 5 | 5 | 4 | 2.268 | 0.0859 | ns |
| 5D, SF3D | *HLA-A* mRNA | AEC | UI | MAIT | AEC | *Sp* | MAIT | 5 | 5 | 4 | 3.072 | 0.0372 | * |
| 5E, SF3G | *IRF1* mRNA | AEC | UI | NT | AEC | *Sp* | NT | 5 | 5 | 4 | 2.977 | 0.0409 | * |
| 5E, SF3G | *IRF1* mRNA | AEC | UI | NT | AEC | UI | MAIT | 5 | 5 | 4 | 4.702 | 0.0093 | ** |
| 5E, SF3G | *IRF1* mRNA | AEC | UI | NT | AEC | *Sp* | MAIT | 5 | 5 | 4 | 4.822 | 0.0085 | ** |
| 5E, SF3G | *IRF1* mRNA | AEC | *Sp* | NT | AEC | UI | MAIT | 5 | 5 | 4 | 3.392 | 0.0275 | * |
| 5E, SF3G | *IRF1* mRNA | AEC | *Sp* | NT | AEC | *Sp* | MAIT | 5 | 5 | 4 | 4.703 | 0.0093 | ** |
| 5E, SF3G | *IRF1* mRNA | AEC | UI | MAIT | AEC | *Sp* | MAIT | 5 | 5 | 4 | 3.813 | 0.0189 | * |
| 5F, SF3C | *NLRC5* mRNA | AEC | UI | NT | AEC | *Sp* | NT | 5 | 5 | 4 | 2.084 | 0.1055 | ns |
| 5F, SF3C | *NLRC5* mRNA | AEC | UI | NT | AEC | UI | MAIT | 5 | 5 | 4 | 3.605 | 0.0227 | * |
| 5F, SF3C | *NLRC5* mRNA | AEC | UI | NT | AEC | *Sp* | MAIT | 5 | 5 | 4 | 4.933 | 0.0079 | ** |
| 5F, SF3C | *NLRC5* mRNA | AEC | *Sp* | NT | AEC | UI | MAIT | 5 | 5 | 4 | 0.6506 | 0.5508 | ns |
| 5F, SF3C | *NLRC5* mRNA | AEC | *Sp* | NT | AEC | *Sp* | MAIT | 5 | 5 | 4 | 3.829 | 0.0186 | * |
| 5F, SF3C | *NLRC5* mRNA | AEC | UI | MAIT | AEC | *Sp* | MAIT | 5 | 5 | 4 | 5.008 | 0.0074 | ** |
| 6A, SF3E | *HLA-A* mRNA | B2B | UI | NT | B2B | *Ms* | NT | 3 | 3 | 2 | 0.09115 | 0.9357 | ns |
| 6A, SF3E | *HLA-A* mRNA | B2B | UI | NT | B2B | UI | MAIT | 3 | 3 | 2 | 2.963 | 0.0975 | ns |
| 6A, SF3E | *HLA-A* mRNA | B2B | UI | NT | B2B | *Ms* | MAIT | 3 | 3 | 2 | 5.839 | 0.0281 | * |
| 6A, SF3E | *HLA-A* mRNA | B2B | *Ms* | NT | B2B | UI | MAIT | 3 | 3 | 2 | 3.015 | 0.0946 | ns |
| 6A, SF3E | *HLA-A* mRNA | B2B | *Ms* | NT | B2B | *Ms* | MAIT | 3 | 3 | 2 | 5.658 | 0.0298 | * |
| 6A, SF3E | *HLA-A* mRNA | B2B | UI | MAIT | B2B | *Ms* | MAIT | 3 | 3 | 2 | 4.79 | 0.0409 | * |
| 6B, SF3F | *HLA-A* mRNA | B2B | UT | NT | B2B | 5-OP | NT | 3 | 3 | 2 | 0.3951 | 0.7309 | ns |
| 6B, SF3F | *HLA-A* mRNA | B2B | UT | NT | B2B | UT | MAIT | 3 | 3 | 2 | 2.963 | 0.0975 | ns |
| 6B, SF3F | *HLA-A* mRNA | B2B | UT | NT | B2B | 5-OP | MAIT | 3 | 3 | 2 | 11.72 | 0.0072 | ** |
| 6B, SF3F | *HLA-A* mRNA | B2B | 5-OP | NT | B2B | UT | MAIT | 3 | 3 | 2 | 3.413 | 0.0762 | ns |
| 6B, SF3F | *HLA-A* mRNA | B2B | 5-OP | NT | B2B | 5-OP | MAIT | 3 | 3 | 2 | 10.2 | 0.0095 | ** |
| 6B, SF3F | *HLA-A* mRNA | B2B | UT | MAIT | B2B | 5-OP | MAIT | 3 | 3 | 2 | 10.94 | 0.0083 | ** |
| 6B, SF3F | *HLA-A* mRNA | B2B | UT | NT | B2B | 6-FP | NT | 3 | 3 | 2 | 0.1102 | 0.9223 | ns |
| 6B, SF3F | *HLA-A* mRNA | B2B | UT | NT | B2B | 6-FP | MAIT | 3 | 3 | 2 | 2.58 | 0.1231 | ns |
| 6B, SF3F | *HLA-A* mRNA | B2B | 6-FP | NT | B2B | UT | MAIT | 3 | 3 | 2 | 2.7 | 0.1142 | ns |
| 6B, SF3F | *HLA-A* mRNA | B2B | 6-FP | NT | B2B | 6-FP | MAIT | 3 | 3 | 2 | 2.416 | 0.1370 | ns |
| 6B, SF3F | *HLA-A* mRNA | B2B | UT | MAIT | B2B | 6-FP | MAIT | 3 | 3 | 2 | 0.3708 | 0.7464 | ns |
| 6B, SF3F | *HLA-A* mRNA | B2B | 5-OP | NT | B2B | 6-FP | NT | 3 | 3 | 2 | 0.4784 | 0.6796 | ns |
| 6C, SF3H | *IRF1* mRNA | B2B | UI | NT | B2B | *Ms* | NT | 3 | 3 | 2 | 0.5695 | 0.6264 | ns |
| 6C, SF3H | *IRF1* mRNA | B2B | UI | NT | B2B | UI | MAIT | 3 | 3 | 2 | 2.556 | 0.125 | ns |
| 6C, SF3H | *IRF1* mRNA | B2B | UI | NT | B2B | *Ms* | MAIT | 3 | 3 | 2 | 7.917 | 0.0156 | * |
| 6C, SF3H | *IRF1* mRNA | B2B | *Ms* | NT | B2B | UI | MAIT | 3 | 3 | 2 | 2.662 | 0.1169 | ns |
| 6C, SF3H | *IRF1* mRNA | B2B | *Ms* | NT | B2B | *Ms* | MAIT | 3 | 3 | 2 | 7.927 | 0.0155 | * |
| 6C, SF3H | *IRF1* mRNA | B2B | UI | MAIT | B2B | *Ms* | MAIT | 3 | 3 | 2 | 7.303 | 0.0182 | * |
| 6D, SF3I | *IRF1* mRNA | B2B | UT | NT | B2B | 5-OP | NT | 3 | 3 | 2 | 1.442 | 0.2860 | ns |
| 6D, SF3I | *IRF1* mRNA | B2B | UT | NT | B2B | UT | MAIT | 3 | 3 | 2 | 2.556 | 0.1250 | ns |
| 6D, SF3I | *IRF1* mRNA | B2B | UT | NT | B2B | 5-OP | MAIT | 3 | 3 | 2 | 7.652 | 0.0167 | * |
| 6D, SF3I | *IRF1* mRNA | B2B | 5-OP | NT | B2B | UT | MAIT | 3 | 3 | 2 | 2.693 | 0.1147 | ns |
| 6D, SF3I | *IRF1* mRNA | B2B | 5-OP | NT | B2B | 5-OP | MAIT | 3 | 3 | 2 | 7.67 | 0.0166 | * |
| 6D, SF3I | *IRF1* mRNA | B2B | UT | MAIT | B2B | 5-OP | MAIT | 3 | 3 | 2 | 8.056 | 0.0151 | * |
| 6D, SF3I | *IRF1* mRNA | B2B | UT | NT | B2B | 6-FP | NT | 3 | 3 | 2 | 0.212 | 0.8518 | ns |
| 6D, SF3I | *IRF1* mRNA | B2B | UT | NT | B2B | 6-FP | MAIT | 3 | 3 | 2 | 2.399 | 0.1385 | ns |
| 6D, SF3I | *IRF1* mRNA | B2B | 6-FP | NT | B2B | UT | MAIT | 3 | 3 | 2 | 2.539 | 0.1263 | ns |
| 6D, SF3I | *IRF1* mRNA | B2B | 6-FP | NT | B2B | 6-FP | MAIT | 3 | 3 | 2 | 2.392 | 0.1392 | ns |
| 6D, SF3I | *IRF1* mRNA | B2B | UT | MAIT | B2B | 6-FP | MAIT | 3 | 3 | 2 | 3.075 | 0.0915 | ns |
| 6D, SF3I | *IRF1* mRNA | B2B | 5-OP | NT | B2B | 6-FP | NT | 3 | 3 | 2 | 1.361 | 0.3065 | ns |

*Definition of abbreviations:*

Ag = antigen; df = degrees of freedom; statistic = absolute value of T statistic; AEC = airway epithelial cells; B2B = BEAS-2B cells; UI = uninfected control; *Sp* = *Streptococcus pneumoniae*; *Ms* = *Mycobacterium smegmatis*; UT = media treated control; 5-OP = 5-OP-RU; 6-FP = 6-formylpterin; NT = no T cell control. Sig: **** for p<0.0001; *** for 0.0001<p< 0.001; ** for 0.001<p< 0.01; * for 0.01<p< 0.05; ns for p>0.05.
