# Supplementary material for: IFNγ regulates MR1 transcription and antigen presentation
Source: Front Immunol. 2025 Sep 26;16:1624767. doi: 10.3389/fimmu.2025.1624767 (PMC12510863; doi:10.3389/fimmu.2025.1624767)
Supplement: Supplementary file 8 [file Table4.docx]

**Supplementary Table 4 Statistics associated with Figure 6E-F.**

| Figure | **Sample 1** | | | **Sample 2** | | | n1 | n2 | df | statistic | p-value | sig. |
| --- | --- | --- | --- | --- | --- | --- | --- | --- | --- | --- | --- | --- |
|  | Cell | Ag | T cell | Cell | Ag | T cell |  |  |  |  |  |  |
| 6E | Cas9 | UI | NT | Cas9 | *Ms* | NT | 3 | 3 | 2 | 1.2016 | 0.3525 | ns |
| 6E | Cas9 | UI | NT | Cas9 | UI | MAIT | 3 | 3 | 2 | 2.6842 | 0.1153 | ns |
| 6E | Cas9 | UI | NT | Cas9 | *Ms* | MAIT | 3 | 3 | 2 | 17.538 | 0.0032 | ** |
| 6E | Cas9 | *Ms* | NT | Cas9 | UI | MAIT | 3 | 3 | 2 | 0.9623 | 0.4374 | ns |
| 6E | Cas9 | *Ms* | NT | Cas9 | *Ms* | MAIT | 3 | 3 | 2 | 16.509 | 0.0037 | ** |
| 6E | Cas9 | UI | MAIT | Cas9 | *Ms* | MAIT | 3 | 3 | 2 | 21.563 | 0.0021 | ** |
| 6E | IRF1 KO #1 | UI | NT | IRF1 KO #1 | *Ms* | NT | 3 | 3 | 2 | 1.6008 | 0.2506 | ns |
| 6E | IRF1 KO #1 | UI | NT | IRF1 KO #1 | UI | MAIT | 3 | 3 | 2 | 5.4716 | 0.0318 | * |
| 6E | IRF1 KO #1 | UI | NT | IRF1 KO #1 | *Ms* | MAIT | 3 | 3 | 2 | 13.345 | 0.0056 | ** |
| 6E | IRF1 KO #1 | *Ms* | NT | IRF1 KO #1 | UI | MAIT | 3 | 3 | 2 | 5.1824 | 0.0353 | * |
| 6E | IRF1 KO #1 | *Ms* | NT | IRF1 KO #1 | *Ms* | MAIT | 3 | 3 | 2 | 11.169 | 0.0079 | ** |
| 6E | IRF1 KO #1 | UI | MAIT | IRF1 KO #1 | *Ms* | MAIT | 3 | 3 | 2 | 3.9011 | 0.0599 | ns |
| 6F | Cas9 | UT | NT | Cas9 | 5-OP | NT | 3 | 3 | 2 | 1.3995 | 0.2966 | ns |
| 6F | Cas9 | UT | NT | Cas9 | UT | MAIT | 3 | 3 | 2 | 1.3026 | 0.3225 | ns |
| 6F | Cas9 | UT | NT | Cas9 | 5-OP | MAIT | 3 | 3 | 2 | 2.967 | 0.0973 | ns |
| 6F | Cas9 | 5-OP | NT | Cas9 | UT | MAIT | 3 | 3 | 2 | 1.29093 | 0.3258 | ns |
| 6F | Cas9 | 5-OP | NT | Cas9 | 5-OP | MAIT | 3 | 3 | 2 | 3.4192 | 0.0759 | ns |
| 6F | Cas9 | UT | MAIT | Cas9 | 5-OP | MAIT | 3 | 3 | 2 | 3.0747 | 0.0915 | ns |
| 6F | IRF1 KO #1 | UT | NT | IRF1 KO #1 | 5-OP | NT | 3 | 3 | 2 | 1.7513 | 0.2220 | ns |
| 6F | IRF1 KO #1 | UT | NT | IRF1 KO #1 | UT | MAIT | 3 | 3 | 2 | 2.4937 | 0.1302 | ns |
| 6F | IRF1 KO #1 | UT | NT | IRF1 KO #1 | 5-OP | MAIT | 3 | 3 | 2 | 3.0603 | 0.0922 | ns |
| 6F | IRF1 KO #1 | 5-OP | NT | IRF1 KO #1 | UT | MAIT | 3 | 3 | 2 | 2.0063 | 0.1827 | ns |
| 6F | IRF1 KO #1 | 5-OP | NT | IRF1 KO #1 | 5-OP | MAIT | 3 | 3 | 2 | 2.3991 | 0.1385 | ns |
| 6F | IRF1 KO #1 | UT | MAIT | IRF1 KO #1 | 5-OP | MAIT | 3 | 3 | 2 | 1.9399 | 0.1919 | ns |

*Definition of abbreviations:*

Ag = antigen; df = degrees of freedom; statistic = absolute value of T statistic; Cas9 = Cas9^+^ BEAS-2B control cells; IRF1 KO = CRISPR gene knockout cell line; UI = uninfected control; *Ms* = *Mycobacterium smegmatis*; UT = media treated control; 5-OP = 5-OP-RU; NT = no T cell control. Sig: **** for p<0.0001; *** for 0.0001<p< 0.001; ** for 0.001<p< 0.01; * for 0.01<p< 0.05; ns for p>0.05.
