# Supplementary material for: IFNγ regulates MR1 transcription and antigen presentation
Source: Front Immunol. 2025 Sep 26;16:1624767. doi: 10.3389/fimmu.2025.1624767 (PMC12510863; doi:10.3389/fimmu.2025.1624767)
Supplement: Supplementary file 9 [file Table5.docx]

**Supplementary Table 5 Statistics associated with Figure 7 and Supplemental Figure 4.**

| Fig. | Data | Sample 1 | Sample 2 | n1 | n2 | df | statistic | p-value | sig. | test |
| --- | --- | --- | --- | --- | --- | --- | --- | --- | --- | --- |
| 7A | *MR1* mRNA | UT | IFNβ | 8 | 5 | 4 | 6.185 | 0.003472 | ** | meANOVA |
| 7A | *MR1* mRNA | UT | IFNγ | 8 | 5 | 4 | 21.57 | 0.000027 | **** | meANOVA |
| 7A | *MR1* mRNA | UT | IFNλ | 8 | 5 | 4 | 0.4827 | 0.654558 | ns | meANOVA |
| 7A | *MR1* mRNA | UT | TNFα | 8 | 3 | 2 | 3.477 | 0.073706 | ns | meANOVA |
| 7A | *MR1* mRNA | UT | IL-17 | 8 | 4 | 3 | 0.5318 | 0.631732 | ns | meANOVA |
| 7A | *MR1* mRNA | IFNβ | IFNγ | 5 | 5 | 5.826 | 3.964 | 0.007872 | ** | meANOVA |
| 7A | *MR1* mRNA | IFNβ | IFNλ | 5 | 5 | 6.178 | 5.202 | 0.001839 | ** | meANOVA |
| 7A | *MR1* mRNA | IFNβ | TNFα | 5 | 3 | 4.141 | 6.591 | 0.002428 | ** | meANOVA |
| 7A | *MR1* mRNA | IFNβ | IL-17 | 5 | 4 | 5.213 | 5.938 | 0.001675 | ** | meANOVA |
| 7A | *MR1* mRNA | IFNγ | IFNλ | 5 | 5 | 7.918 | 14.1 | 0.000001 | **** | meANOVA |
| 7A | *MR1* mRNA | IFNγ | TNFα | 5 | 3 | 4.562 | 21.73 | 0.000009 | **** | meANOVA |
| 7A | *MR1* mRNA | IFNγ | IL-17 | 5 | 4 | 6.977 | 17.04 | 0.000001 | **** | meANOVA |
| 7A | *MR1* mRNA | IFNλ | TNFα | 5 | 3 | 4.463 | 1.297 | 0.257655 | ns | meANOVA |
| 7A | *MR1* mRNA | IFNλ | IL-17 | 5 | 4 | 6.838 | 0.7041 | 0.504628 | ns | meANOVA |
| 7A | *MR1* mRNA | TNFα | IL-17 | 3 | 4 | 3.633 | 0.5922 | 0.588597 | ns | meANOVA |
| SF4A | *HLA-A* mRNA | UT | IFNβ | 6 | 5 | 4 | 6.849 | 0.002378 | ** | meANOVA |
| SF4A | *HLA-A* mRNA | UT | IFNγ | 6 | 5 | 4 | 9.193 | 0.000778 | *** | meANOVA |
| SF4A | *HLA-A* mRNA | UT | IFNλ | 6 | 4 | 3 | 2.529 | 0.085468 | ns | meANOVA |
| SF4A | *HLA-A* mRNA | UT | TNFα | 6 | 3 | 2 | 3.306 | 0.080582 | ns | meANOVA |
| SF4A | *HLA-A* mRNA | UT | IL-17 | 6 | 3 | 2 | 3.613 | 0.068792 | ns | meANOVA |
| SF4A | *HLA-A* mRNA | IFNβ | IFNγ | 5 | 5 | 7.344 | 0.07737 | 0.940398 | ns | meANOVA |
| SF4A | *HLA-A* mRNA | IFNβ | IFNλ | 5 | 4 | 4.763 | 5.78 | 0.002559 | ** | meANOVA |
| SF4A | *HLA-A* mRNA | IFNβ | TNFα | 5 | 3 | 5.873 | 3.196 | 0.01925 | * | meANOVA |
| SF4A | *HLA-A* mRNA | IFNβ | IL-17 | 5 | 3 | 4.151 | 6.29 | 0.002878 | ** | meANOVA |
| SF4A | *HLA-A* mRNA | IFNγ | IFNλ | 5 | 4 | 5.352 | 7.464 | 0.000505 | *** | meANOVA |
| SF4A | *HLA-A* mRNA | IFNγ | TNFα | 5 | 3 | 4.911 | 3.667 | 0.014962 | * | meANOVA |
| SF4A | *HLA-A* mRNA | IFNγ | IL-17 | 5 | 3 | 4.278 | 8.366 | 0.000829 | *** | meANOVA |
| SF4A | *HLA-A* mRNA | IFNλ | TNFα | 4 | 3 | 2.589 | 2.187 | 0.130748 | ns | meANOVA |
| SF4A | *HLA-A* mRNA | IFNλ | IL-17 | 4 | 3 | 4.051 | 0.8558 | 0.43978 | ns | meANOVA |
| SF4A | *HLA-A* mRNA | TNFα | IL-17 | 3 | 3 | 2.113 | 2.66 | 0.110489 | ns | meANOVA |
| SF4B | *IRF1* mRNA | UT | IFNβ | 7 | 5 | 4 | 16.03 | 0.000089 | **** | meANOVA |
| SF4B | *IRF1* mRNA | UT | IFNγ | 7 | 5 | 4 | 17.41 | 0.000064 | **** | meANOVA |
| SF4B | *IRF1* mRNA | UT | IFNλ | 7 | 5 | 4 | 5.514 | 0.005281 | ** | meANOVA |
| SF4B | *IRF1* mRNA | UT | TNFα | 7 | 3 | 2 | 17.05 | 0.003424 | ** | meANOVA |
| SF4B | *IRF1* mRNA | UT | IL-17 | 7 | 4 | 3 | 0.5277 | 0.634268 | ns | meANOVA |
| SF4B | *IRF1* mRNA | IFNβ | IFNγ | 5 | 5 | 4.144 | 15.12 | 0.000088 | **** | meANOVA |
| SF4B | *IRF1* mRNA | IFNβ | IFNλ | 5 | 5 | 5.551 | 12.36 | 0.00003 | **** | meANOVA |
| SF4B | *IRF1* mRNA | IFNβ | TNFα | 5 | 3 | 4.824 | 9.799 | 0.00023 | *** | meANOVA |
| SF4B | *IRF1* mRNA | IFNβ | IL-17 | 5 | 4 | 4.044 | 15.95 | 0.000084 | **** | meANOVA |
| SF4B | *IRF1* mRNA | IFNγ | IFNλ | 5 | 5 | 4.029 | 17.05 | 0.000066 | **** | meANOVA |
| SF4B | *IRF1* mRNA | IFNγ | TNFα | 5 | 3 | 4.016 | 16.63 | 0.000074 | **** | meANOVA |
| SF4B | *IRF1* mRNA | IFNγ | IL-17 | 5 | 4 | 4.001 | 17.41 | 0.000064 | **** | meANOVA |
| SF4B | *IRF1* mRNA | IFNλ | TNFα | 5 | 3 | 5.986 | 5.755 | 0.001209 | ** | meANOVA |
| SF4B | *IRF1* mRNA | IFNλ | IL-17 | 5 | 4 | 4.217 | 5.354 | 0.005049 | ** | meANOVA |
| SF4B | *IRF1* mRNA | TNFα | IL-17 | 3 | 4 | 2.198 | 16.53 | 0.002389 | ** | meANOVA |
| SF4C | *B2m* mRNA | UT | IFNβ | 6 | 5 | 4 | 6.381 | 0.003094 | ** | meANOVA |
| SF4C | *B2m* mRNA | UT | IFNγ | 6 | 4 | 3 | 12.02 | 0.001238 | ** | meANOVA |
| SF4C | *B2m* mRNA | UT | IFNλ | 6 | 5 | 4 | 7.142 | 0.002033 | ** | meANOVA |
| SF4C | *B2m* mRNA | UT | TNFα | 6 | 3 | 2 | 2.596 | 0.121832 | ns | meANOVA |
| SF4C | *B2m* mRNA | UT | IL-17 | 6 | 4 | 3 | 0.437 | 0.691667 | ns | meANOVA |
| SF4C | *B2m* mRNA | IFNβ | IFNγ | 5 | 4 | 5.131 | 1.619 | 0.164846 | ns | meANOVA |
| SF4C | *B2m* mRNA | IFNβ | IFNλ | 5 | 5 | 4.273 | 4.976 | 0.006405 | ** | meANOVA |
| SF4C | *B2m* mRNA | IFNβ | TNFα | 5 | 3 | 4.912 | 5.148 | 0.003809 | ** | meANOVA |
| SF4C | *B2m* mRNA | IFNβ | IL-17 | 5 | 4 | 4.046 | 6.33 | 0.003067 | ** | meANOVA |
| SF4C | *B2m* mRNA | IFNγ | IFNλ | 4 | 5 | 4.361 | 7.76 | 0.001037 | ** | meANOVA |
| SF4C | *B2m* mRNA | IFNγ | TNFα | 4 | 3 | 4.934 | 7.108 | 0.000902 | *** | meANOVA |
| SF4C | *B2m* mRNA | IFNγ | IL-17 | 4 | 4 | 3.232 | 11.71 | 0.000934 | *** | meANOVA |
| SF4C | *B2m* mRNA | IFNλ | TNFα | 5 | 3 | 3.124 | 1.005 | 0.386337 | ns | meANOVA |
| SF4C | *B2m* mRNA | IFNλ | IL-17 | 5 | 4 | 5.267 | 6.439 | 0.001104 | ** | meANOVA |
| SF4C | *B2m* mRNA | TNFα | IL-17 | 3 | 4 | 2.186 | 2.447 | 0.123586 | ns | meANOVA |
| 7B | *MR1* mRNA | UT | IFNβ | 5 | 5 | 4 | 6.185 | 0.003472 | ** | paired t test |
| 7B | *MR1* mRNA | UT | IFNγ | 5 | 5 | 4 | 21.57 | 0.000027 | **** | paired t test |
| 7B | *MR1* mRNA | IFNγ | IFNβ | 3 | 3 | 2 | 3.964 | 0.007872 | ** | paired t test |
| 7C | *HLA-A* mRNA | UT | IFNβ | 5 | 5 | 4 | 6.849 | 0.002378 | ** | paired t test |
| 7C | *HLA-A* mRNA | UT | IFNγ | 5 | 5 | 4 | 9.193 | 0.000778 | *** | paired t test |
| 7C | *HLA-A* mRNA | IFNγ | IFNβ | 4 | 4 | 3 | 0.07737 | 0.940398 | ns | paired t test |
| 7D | αMR1 gMFI | UT | IFNβ | 5 | 5 | 4 | 6.403 | 0.003056 | ** | paired t test |
| 7D | αMR1 gMFI | UT | IFNγ | 5 | 5 | 4 | 2.032 | 0.111905 | ns | paired t test |
| 7D | αMR1 gMFI | IFNγ | IFNβ | 5 | 5 | 4 | 10.22 | 0.000517 | *** | paired t test |
| 7D | αMHC-Ia gMFI | UT | IFNβ | 5 | 5 | 4 | 31.44 | 0.000006 | **** | paired t test |
| 7D | αMHC-Ia gMFI | UT | IFNγ | 5 | 5 | 4 | 13.56 | 0.000171 | *** | paired t test |
| 7D | αMHC-Ia gMFI | IFNγ | IFNβ | 5 | 5 | 4 | 3.681 | 0.021184 | * | paired t test |
| 7F | *IRF1* mRNA | UT | IFNβ | 5 | 5 | 4 | 16.03 | 0.000089 | **** | paired t test |
| 7F | *IRF1* mRNA | UT | IFNγ | 5 | 5 | 4 | 17.41 | 0.000064 | **** | paired t test |
| 7F | *IRF1* mRNA | IFNγ | IFNβ | 4 | 4 | 3 | 15.12 | 0.000088 | **** | paired t test |
| 7G | *NLRC5* mRNA | UT | IFNβ | 3 | 3 | 2 | 5.159 | 0.035586 | * | paired t test |
| 7G | *NLRC5* mRNA | UT | IFNγ | 4 | 4 | 3 | 9.844 | 0.002229 | ** | paired t test |
| 7G | *NLRC5* mRNA | IFNγ | IFNβ | 3 | 3 | 2 | 2.37 | 0.124521 | ns | paired t test |
| 7H | *B2m* mRNA | UT | IFNβ | 5 | 5 | 4 | 6.381 | 0.003094 | ** | paired t test |
| 7H | *B2m* mRNA | UT | IFNγ | 4 | 4 | 4 | 12.02 | 0.001238 | ** | paired t test |
| 7H | *B2m* mRNA | IFNγ | IFNβ | 4 | 4 | 3 | 1.619 | 0.164846 | ns | paired t test |

*Definition of abbreviations:*

df = degrees of freedom; statistic = absolute value of statistic; UT = media treated control; meANOVA = Mixed-effects Analysis of Variance. Sig: **** for p<0.0001; *** for 0.0001<p< 0.001; ** for 0.001<p< 0.01; * for 0.01<p< 0.05; ns for p>0.05.
